# Supplementary material for: ATOMS (Adjustable Trans-Obturator Male System) in Patients with Post-Prostatectomy Incontinence and Previously Treated Urethral Stricture or Bladder Neck Contracture
Source: J Clin Med. 2022 Aug 19;11(16):4882. doi: 10.3390/jcm11164882 (PMC9410097; doi:10.3390/jcm11164882)
Supplement: Supplementary file 1 [file jcm-11-04882-s001.zip › jcm-1822672-supplementary.pdf]

SUPPLEMENTARY MATERIAL

Table S1. Main characteristics of the 21 patients implanted with ATOMS and previous treatment of urethral stricture or bladder neck contracture.

Patient number	Previous Stricture location	Type of intervention	Previous radiation	Severity of SUI	ICIQ-SF	Baseline 24-h pad-test (ml)	Age at ATOMS implant (years)	Number of adjustments	Final cushion volume (ml)	Post ATOMS 24-h pad-test (ml)
5	BLADDER NECK	BLADDER NECK INCISION	YES	SEVERE	21	900	78	3	30	40
8	BULBO-MEMBRANOUS	URETHROPLASTY	YES	SEVERE	14	480	69	5	37	450
22	BLADDER NECK	BLADDER NECK INCISION	NO	SEVERE	19	1225	64	3	21	5
30	BLADDER NECK	BLADDER NECK INCISION	NO	SEVERE	20	1200	64	3	24	20
50	PENDULOUS	DVIU	NO	SEVERE	19	1200	73	3	26	230
54	BULBAR	DVIU	NO	SEVERE	14	650	65	2	16	0
60	PENDULOUS	DVIU	NO	MODERATE	14	648	67	2	17	90
62	BULBAR	URETHROPLASTY	YES	SEVERE	21	1800	73	4	23.5	280
81	BULBO-MEMBRANOUS	URETHROPLASTY	NO	SEVERE	19	980	65	3	17	70
82	BULBAR	URETHROPLASTY	NO	SEVERE	21	1600	71	3	21.5	90
90	BULBO-MEMBRANOUS	DVIU	NO	MODERATE	16	450	68	2	14	0
91	BULBAR	DVIU	NO	SEVERE	16	680	78	4	20.5	0
96	BULBO-MEMBRANOUS	URETHROPLASTY	YES	MODERATE	18	635	70	2	14	0
99	BULBO-MEMBRANOUS	URETHROPLASTY	NO	SEVERE	20	950	65	3	21	120
100	BULBO-MEMBRANOUS	URETHROPLASTY	YES	SEVERE	18	650	66	2	16.5	0
101	PENDULOUS	DVIU	NO	MODERATE	21	400	58	1	14	0
112	BLADDER NECK	BLADDER NECK INCISION	YES	SEVERE	18	1480	83	2	17	680
114	BULBO-MEMBRANOUS	DVIU	YES	SEVERE	21	1200	71	3	16	110
116	BULBO-MEMBRANOUS	DVIU	YES	SEVERE	21	980	78	3	16	180
127	PENDULOUS	DVIU	NO	SEVERE	14	650	65	1	14	0
133	BLADDER NECK	BLADDER NECK INCISION	YES	SEVERE	21	1300	69	3	16	180
